# *Cupiennius* spiders (Trechaleidae) from southern Mexico: DNA barcoding, venomics, and biological effect

**DOI:** 10.1590/1678-9199-JVATITD-2023-0098

**Published:** 2024-08-12

**Authors:** Montserrat Padilla-Villavicencio, Gerardo Corzo, Karina Guillén-Navarro, Guillermo Ibarra-Núñez, Iván Arenas, Fernando Zamudio, Elia Diego-García

**Affiliations:** 1 El Colegio de la Frontera Sur (ECOSUR), Grupo Académico de Biotecnología Ambiental, Tapachula, Chiapas, Mexico.; 2 Departamento de Medicina Molecular, Instituto de Biotecnología, Universidad Nacional Autónoma de México (Unam), Cuernavaca, Morelos, Mexico.; 3 El Colegio de la Frontera Sur (ECOSUR), Colección de Arácnidos del Sureste de México, Grupo Académico de Biología y Ecología de Artrópodos Benéficos, Tapachula, Chiapas, Mexico.; 4 Programa Investigadoras e Investigadores por México del CONAHCyT - El Colegio de la Frontera, Mexico City, Mexico.

**Keywords:** Cupiennius, Spider toxin, Venom, COI, DNA barcoding.

## Abstract

**Background:**

Members of the genus *Cupiennius* Simon, 1891 are categorized as wandering spiders and are part of the family Trechaleidae. The genomics and proteomics of *Cupiennius* spiders from North America remain uncharacterized. The present study explores for the first time molecular data from the endemic species *Cupiennius chiapanensis* Medina, 2006, and also presents new data for *Cupiennius salei* (Keyserling, 1878), both collected in southern Mexico.

**Methods:**

In total, 88 *Cupiennius* specimens were collected from southern Mexico and morphologically identified. DNA was extracted and the mitochondrial COI fragment was amplified. COI sequences were analyzed, and a phylogenetic tree was inferred for species from the Americas. Genetic diversity was analyzed using haplotype networks and gene distances. Venom was obtained from *C. chiapanensis* and *C. salei* by electrostimulation. The venom was separated by HPLC, visualized using SDS-PAGE, and quantified for use in toxicity bioassays in mice and insects.

**Results:**

Analysis of COI sequences from *C. chiapanensis* showed 94% identity with *C. salei*, while *C. salei* exhibited 94-97% identity with sequences from Central and South American conspecifics. The venom from *C. chiapanensis* exhibited toxic activity against crickets. Venoms from *C. chiapanensis* and *C. salei* caused death in *Anastrepha obliqua* flies. Analysis of venom fractions from *C. salei* and *C. chiapanensis* revealed molecular masses of a similar size as some previously reported toxins and neurotoxic components. We determined the amino acid sequences of ChiaTx1 and ChiaTx2, toxins that are reported here for the first time and which showed toxicity against mice and insects.

**Conclusion:**

Our work is the first to report COI-based DNA barcoding sequences from southern Mexican *Cupiennius* spiders. Compounds with toxic activity were identified in venom from both species.

## Background

Spiders are widely distributed over the world, with more than 52,000 described species so far [1]. The last decade witnessed major advances in the analysis of phylogenetic data, which has allowed us to resolve and understand phylogenetic relationships using molecular markers (such as nuclear and ribosomal genes) [2, 3]. The genus *Cupiennius* Simon, 1891 is placed in the family Trechaleidae Simon, 1890, which ranks 60th in spider diversity globally and includes 133 species grouped in 17 genera [1]. *Cupiennius* currently consists of 11 species. These species are distributed from southern Mexico to South America and the West Indies [1]. *Cupiennius* belongs to the group of wandering spiders and its members do not construct webs but ambush their prey. Moreover, they are generalists and feed on a wide variety of organisms [4], including insects and even some small vertebrates [5]. *Cupiennius* spiders are generally nocturnal predators and live on plants, especially monocotyledonous plants such as bromeliads and bananas, among others [6].

*Cupiennius chiapanensis* was first described in 2006 from the *La Encrucijada* Biosphere Reserve, a mangrove forest in Chiapas, southern Mexico [7]. It is considered an endemic species and is distinguished from other *Cupiennius* species by the color of its chelicerae, which are covered by bright red and pale red hairs in females and males, respectively, and by details of their genital structures [7]. No additional biology or distribution data have entered the published literature since its description was published. 

*Cupiennius salei*, on the other hand, was described in 1877 [8], and its current distribution covers parts of Mexico, Central America, and Hispaniola [1, 9]. Since the 1960s, extensive studies using specimens from Central America have produced a comprehensive body of knowledge, incorporating findings from the study of the sensory system, functional morphology, and species biology and behavior [6]. Kuhn-Nentwig and collaborators have published several studies following the discovery of the CSTX-1 toxin in 1994 [10]. CSTX-1 is the most abundant neurotoxin in venom from *C. salei* and was found to possess the highest toxic activity against insects. They reported the analysis of the venom gland transcriptome of *C. salei* and detected various toxin-encoding transcripts [11, 12]. These publications facilitated the characterization of the various toxin families from the venom of *C. salei* and helped us gain insight into the structure and domains of toxins and venom proteins [13, 14, 15]. Components from its venom exhibit a variety of biological activities, for instance cytolytic [16], hyaluronidase [10], and insecticidal activity [12].

In recent decades, the generation of genetic information for several arachnids has advanced the molecular identification of species. A number of phylogenies derived from molecular markers, such as mitochondrial, nuclear, and ribosomal genes, have been proposed [2, 3, 17, 18]. DNA barcoding is a practical tool for the molecular identification of species and typically uses the mitochondrial cytochrome c oxidase subunit I (COI) gene as a molecular marker for animals [19]. Because of its lack of introns, limited exposure to recombination, and the availability of robust primer sites, COI is frequently proposed as a DNA barcoding marker for spiders [20, 2].

For the present study, we collected specimens from different localities across southern Mexico. Total DNA was extracted and the mitochondrial COI fragment was amplified. Data from the DNA barcoding analysis provided insight into the distribution of *Cupiennius* in southern Mexico (Chiapas and Veracruz). Venom recovered from collected specimens was characterized using chromatographic and mass spectrometric techniques to generate a partial mass fingerprint. Purified compounds were bio-assayed for toxic activity against insects and mice.

## Methods

Specimen collection and identification

**Figure 1.**
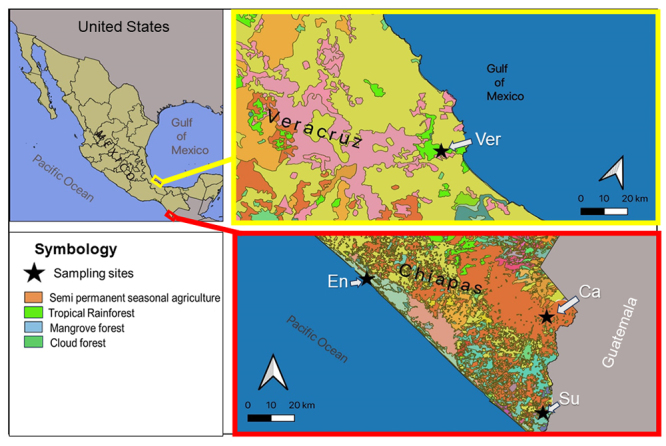
Map of Mexico showing the *Cupiennius* collecting sites. Black stars correspond to the sampling sites in Chiapas: Acapetahua (26 m a.s.l.; 15°12'37" N, 92°53'58.3" W; mangrove forest), Suchiate (33 m a.s.l., 14°38'39" N, 92°11'52" W; banana monoculture) and Cacahoatán (665 m a.s.l., 15°02'18.3" N, 92°10'18.6" W; covered by secondary vegetation); and sampling site in Veracruz: biosphere reserve *La Estación Biológica Los Tuxtlas* is covered by tropical rainforest (150 m a.s.l., 18°35'04" N, 95°04'26" W).

**Figure 2.**
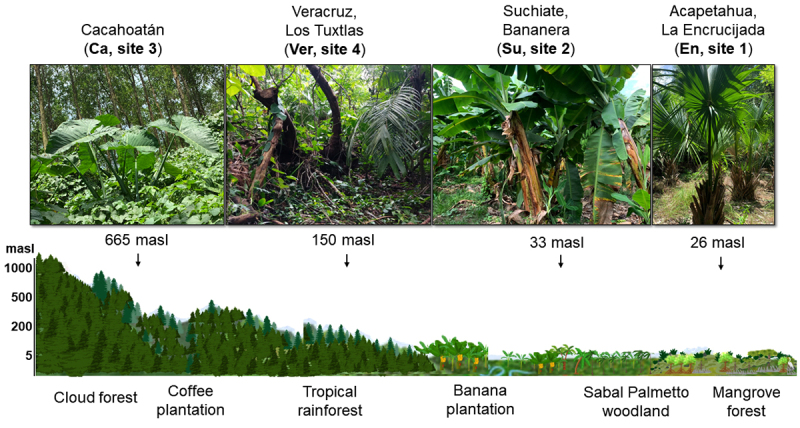
Graphical map showing the *Cupiennius* collecting sites. Site 1 (*La Encrucijada*, En) is in the municipality of Acapetahua (26 m a.s.l.; 15°12'37" N, 92°53'58.3" W; mangrove forest). Site 2 (Suchiate, Su) is in an agricultural area (33 m a.s.l., 14°38'39" N, 92°11'52" W; banana monoculture). The area of Site 3 (Cacahoatán, Ca) is covered by secondary vegetation (665 m a.s.l., 15°02'18.3" N, 92°10'18.6" W) whereas Site 4 (*La Estación Biológica Los Tuxtlas*, Veracruz; Ver) is covered by tropical rainforest (150 m a.s.l., 18°35'04" N, 95°04'26" W).

Sampling sites were located in the municipalities of Cacahoatán, Suchiate, and Acapetahua, Chiapas, southern Mexico. An additional sampling site from Veracruz, southern Mexico, served as a reference site for *C. salei*. Site 1 (*La Encrucijada*, En), in the municipality of Acapetahua near the *La Encrucijada* Biosphere Reserve (26 m a.s.l.; 15°12'37" N, 92°53'58.3" W), is characterized by mangrove forests. Site 2 (Suchiate, Su) is located in an agricultural area of the Soconusco, Chiapas, that is typified by banana monoculture (33 m a.s.l., 14°38'39" N, 92°11'52" W). The selection of this area was based on previous reports that banana plants provide *C. salei* with shelter in Central America [6]. The area of Site 3 (Cacahoatán, Ca) is covered by secondary vegetation (665 m a.s.l., 15°02'18.3" N, 92°10'18.6" W), including coffee and eucalyptus plantations. Site 4 (*La Estación Biológica Los Tuxtlas*, Veracruz; Ver) is located in the State of Veracruz, southern Mexico, and is covered by tropical rainforest (150 m a.s.l., 18°35'04" N, 95°04'26" W). A specimen of *C. salei* was collected from this site as a reference sample because this species had previously been reported from the area (the current reported distribution of *C. salei* in Mexico is limited to Veracruz; see Table 1; Figures 1 and 2).

**Table 1.** Relationship of COI sequences for localities, codes, GenBank accessions, and number of sequences and specimens collected from the genus *Cupiennius* in Mexico.

Origin	Species	Locality	GenBank accession number	Specimen	Latitude (N)	Longitude (W)	Altitude (m)
La Encrucijada (En)	*C. chiapanensis* ♂ ♀	Acapetahua, Chiapas	OR906089 OR906090 OR906091 OR906092	Leg	15°12' 37"	92°53' 58.3"	26
Suchiate (Su)	*C. chiapanensis* ♂ ♀	Suchiate, Chiapas	OR906087 OR906088	Leg	14°38' 39"	92°11' 52"	33
Cacahoat**á**n (Ca)	*C. salei* ♂ ♀	Cacahoatán, Chiapas	OR906093 OR906094	Leg	15°02'18.3"	92°10'18.6"	665
Los Tuxtlas (Ver)	*C. salei* ♀	Los Tuxtlas, Veracruz	OR906095	Leg	18°35' 04"	95°04' 26"	150

Collected specimens were identified based on morphological features using taxonomic keys. For species determination, the specialized literature by Barth & Cordes [21] and Medina [7] was consulted. Coloration patterns and sex (pedipalp and epigynum for males and females, respectively) were determined by observation under a dissecting microscope (Olympus SZX16). Following identification, the specimens were milked to collect their venom. Some specimens were dissected; their legs and venom glands were removed and preserved in RNAlater (Sigma-Aldrich, USA), and stored at -20 °C until DNA extraction.

## DNA isolation and COI amplification

DNA was extracted from one leg of each dissected specimen using the DNeasy Blood & Tissue kit (QIAGEN, UK) following the manufacturer's insect protocol. Extracted DNA was then visualized on 0.8% agarose gel (90 V, 35 minutes) and quantified on a NanoDrop Lite spectrophotometer (Thermo Fisher Scientific, USA).

The COI molecular marker (approximately 720 bp) was amplified by polymerase chain reaction (PCR) using the previously reported primers LCO-1490 (5'- GGTCAACAAATCATAAAGATATTGG-3'; [22]), Chelicerate_R2 (5'-GGATGGCCAAAAAATCAAAATAAATG-3'; [23]), and COIex (5'-CCAGGTAAAATTAAAATATAAACTTC-3'; [24]). A temperature gradient PCR (50 °C to 60 °C) was performed to standardize reaction conditions. PCR conditions were as follows: initial denaturation step of 95 °C for three minutes; followed by 34 cycles of 95 °C for 40 seconds, 60 °C for 40 seconds, 72 °C for one minute; and 72 °C for five minutes. PCR products were purified using the Zymoclean Gel DNA Recovery kit (ZYMO Research, USA) and were sent for Sanger sequencing to Macrogen (Seoul, South Korea) and *La Unidad de Síntesis y Secuenciación de DNA* (USSDNA) at the *Instituto de Biotecnología* of the UNAM (Cuernavaca, Mexico). Alternatively, some products were cloned using pJET1.2 plasmid (Thermo Fisher Scientific, USA) and sent for sequencing. 

## Data processing

DNA sequences from collected *C. chiapanensis* and *C. salei* specimens were analyzed with the BLAST (Basic Local Alignment Search Tool) algorithm [25]. Sequence alignments were constructed using the software programs Clustal Omega 1.2.4 [26] and MEGA 11.0.13 [27]. A data matrix was generated including all sequences from the COI database to estimate genetic distances. The p-distance was calculated using MEGA 11.0.13 [27], and a genetic distance tree was constructed in PAUP v4a [28] using the Kimura 2-parameter model. A median-joining haplotype network (Epsilon = 0) was generated using PopART 1.7 [29] to assess the genetic structure between sampled *C. chiapanensis* and *C. salei* populations. Genetic diversity was assessed by computing the number of haplotypes (h), polymorphic sites (s), nucleotide diversity (π), mean of nucleotide differences (K), nucleotide variation per sequence (θ), and haplotype diversity (Hd) [30, 31, 32]. Partial *C. salei* COI sequences retrieved from databases (GenBank: KM225104.1; BOLD Systems: ACG3675.1) were compared to sequence data generated in the present study. The evolutionary model for COI sequence data was determined using ModelTest-NG [33] under the Bayesian information criterion (BIC). The General Time Reversible model (GTR) with gamma distribution across sites (G4) was selected as the general DNA substitution model. A phylogenetic tree was generated by Bayesian analysis using MrBayes 3.1.2 [34], running four Markov chains using the following parameters: number of Markov Chain Monte Carlo (MCMC) generations = 50,000,000; sample frequency = 500; print frequency = 1,000; number of runs = 2; number of chains = 4. MCMC parameters and effective sample size (ESS) were analyzed using TRACER v1.7 [35] to assess convergence. Tree topology was visualized in the software Figtree 1.4.3 [36], with posterior probability (PP) values indicated on the nodes.

*Phoneutria fera* Perty, 1833 (Ctenidae) (KY017637.1) served as an outgroup. Trechaleid spider COI sequence data included sequences generated in the present study (for *C. chiapanensis* and *C. salei*) as well as sequences retrieved from databases: *C. salei* from French Guyana (KM225104.1; *C. salei_GF*), *C. salei* from Honduras (BOLD:ACG3675; *C. salei_Ho*), *C. granadensis* (Keyserling, 1877) from French Guyana (KY017636.1), and *C. bimaculatus* (Taczanowski, 1874) (OP214418.1). The trechaleid spiders *Trechaleoides biocellata* (Mello-Leitão, 1926) (KY018027.1; [37]) and *Trechaleoides keyserlingi* (F. O. Pickard-Cambridge, 1903) (KY190306.1; [38]) belong to a different genus and were added as additional outgroup species.

## Venom collection

Venom was recovered from all collected *C. chiapanensis* and *C. salei* specimens after identification and within 24 hours after collection. Individuals were milked by electrical stimulation (12 V), and the obtained venom was centrifuged (9,610 xg, 10 minutes). The protein content of the supernatant was quantified using a NanoDrop Lite spectrophotometer (absorbance at 280 nm). Milked individuals remained in captivity and were kept in plastic cages at 25 °C. Specimens were then provided with food, followed by a second milking two weeks later. They were fed with crickets and/or *Anastrepha obliqua* (Macquart, 1835) flies until a second and third milking was carried out under captive conditions.

## Biochemical characterization of the venom

Venom (50 µg) was separated on 12% acrylamide gel under reducing conditions (SDS-PAGE) using Invitrogen SeeBlue Plus2 Pre-stained Protein Standard (Thermo Fisher Scientific, USA) as molecular weight size marker (3-198 kDa). A venom sample (50 μg) was mixed with loading buffer (5% β-mercaptoethanol, 0.5 M Tris pH 6.8, glycerol, SDS 10%, 0.5% bromophenol blue on deionized water). The 12% polyacrylamide gel was run at 100V for 90 minutes, after which the gel was stained for 45 minutes with Bio-Safe Coomassie G-250 Stain (BIO-RAD). The gel was then washed with distilled water for 15 minutes or until observable. As a molecular marker, SeeBlue^TM^ Plus2, Pre-stained Protein Standard (Thermo Fisher Scientific, USA) was used. Venom from the scorpion *Centruroides tapachulaensis* Hoffmann, 1932 and the spider *Davus* aff. *pentaloris* were used as gel electrophoresis references. The spider venom (500-700 μg) was separated and purified by C18 reversed-phase (250 x 4.6 mm, 5 µm, column from Nacalai-Tesque, Japan). High-Performance Liquid Chromatography (HPLC; Agilent Infinity 1260; Agilent, USA) using a gradient elution profile. The mobile phase consisted of 0.1% trifluoroacetic acid (TFA) in water (solvent A) with an eluting solvent of 0.1% TFA in CH_3_CN (solvent B) run over a linear 60 minutes gradient of 0-60% solvent B at a constant flow rate of 1 mL/min. Eluted fractions were monitored at 230 nm. Major fractions were purified again and analyzed by electrospray ionization mass spectrometry (ESI-MS) using an LCQ Fleet ion trap mass spectrometer (Thermo Fisher Scientific, USA). Briefly, the protein fractions were solubilized to a final concentration of 500 pmol/50 mL of 50% acetonitrile with 1% acetic acid and directly applied into a Thermo Scientific LCQ Fleet ion trap mass spectrometer (San José, CA) using a Surveyor MS syringe pump delivery system. The eluate at 10 mL/min was split out to introduce only 5% of the sample into the nanospray source (0.5 mL/min). The spray voltage was set from 1.5 kV and the capillary temperature was set at 150 °C. The fragmentation source was operated at 25-35 V of collision energy, 35-45% (arbitrary units) of normalized collision energy, and the scan with a wide band was activated. All spectra were obtained in the positive-ion mode. The data acquisition and the deconvolution of data were performed on the Xcalibur Windows NT PC data system [39]. The average molecular mass values are ± 1 Da due to the limited resolution of this instrument.

The N-terminal sequences of the purified peptides were obtained by Edman degradation using a PPSQ-31 gas phase protein sequencer (Shimadzu, Japan). The peptide sample (10 µg) was dissolved in 10 µL 37% aqueous CH_3_CN (v/v) and applied to TFA-treated glass fiber membranes pre-cyclized with polybrene (Sigma-Aldrich, USA) at the *Unidad de Proteómica* of the *Instituto de Biotecnología* at the UNAM (Cuernavaca, Mexico).

Peptides were identified by interrogating the UniProtKB database [40], using the keyword “Cupiennius” (April 2024), also taking into consideration previous reports of *C. salei* venom composition.

## Toxic activity against insects and mice

*Toxic activity against adult flies*

The toxic effect of whole venom was tested on adult *Anastrepha obliqua* flies by abdominal injection of the venom (injected doses: 20, 40, and 50 µg in 2 µL of 0.9% saline). Trials consisted of five flies that were injected in the central tergite and were repeated the experiment twice (injected in all doses). As a negative control, a group of five flies was injected with 2 µL saline (0.9% NaCl) (with two replicates). As a positive control, flies were injected with venom from *Centruroides tapachulaensis* (injected doses: 20, 40, and 50 µg in 2 µL of 0.9% saline). For the assay, individuals were placed in glass Petri dishes (Ø 10 cm), properly labeled, and maintained under ambient conditions. Toxic effects were registered as slightly, moderately, or highly toxic if administration led to paralysis after ten, three, or during the first two minutes post-injection, respectively. Lethality was recorded at various time intervals. Individuals were kept under observation for two hours, after which survivors were sacrificed.

## Toxic activity against crickets

Crickets (*Acheta domestica* (Linnaeus, 1758)) weighing 100-300 mg were injected intrathoracically between the second and third pair of legs, with 1 to 2 µg of purified HPLC venom fraction (n = 2 each) per g of cricket using a 10 µL Hamilton microsyringe. Fractions were dissolved in phosphate-buffered saline (PBS) to a final volume of 5 µL. As a negative control, 5 µL PBS was injected, and positive controls were performed with the neurotoxic spider peptide PaluIT2 synthesized in our laboratory [41]. Toxic effects were monitored for 10 minutes following injection and again at 24 hours post-injection.

## Toxic activity against mice

CD-1 mice, weighing 18-20 g, were administered 1 µg of the RP-HPLC collected fractions (n = 2 each) to evaluate their toxic activity. Following the recommendations of Carbone et al. [42], the fractions were injected intracranially. The injection, with a 10 µL micro-syringe fitted with a glass capillary, was performed mid-way between the left eye and the left ear (intracranial). Negative controls were done with dH_2_0 only and positive controls with the neurotoxic scorpion peptide CssII isolated in our laboratory [43]. Activity was monitored for 24 hours post-injection.

## Results

Collection and identification

Of the 88 specimens collected from the four sampling sites, 50 were identified as *C. chiapanensis* and 38 as *C. salei* (Additional file 1). *Cupiennius chiapanensis* was collected from sites 1 and 2 (En and Su, respectively), while *C. salei* was found at sites 3 and 4 (Ca and Ver, respectively).

## DNA barcoding and molecular phylogeny

In total, nine new COI sequences were obtained from *C. chiapanensis* and *C*. *salei*. Sequences were deposited in GenBank (OR906087-OR906095). The morphological identification was corroborated by comparing the sequences (maximum distance among COI sequences = 7.8%), which allowed the spiders to be identified and grouped by site. Sequence analysis for *C. chiapanensis* showed 94% identity with *C. salei* sequences retrieved from databases, while the analysis of sequences from our *C. salei* specimens showed 94-96% identity with database sequences of *C. salei* spiders from Central and South America.

A phylogenetic tree was inferred from the COI sequences obtained in the present study and four sequences retrieved from databases. 

**Figure 3.**
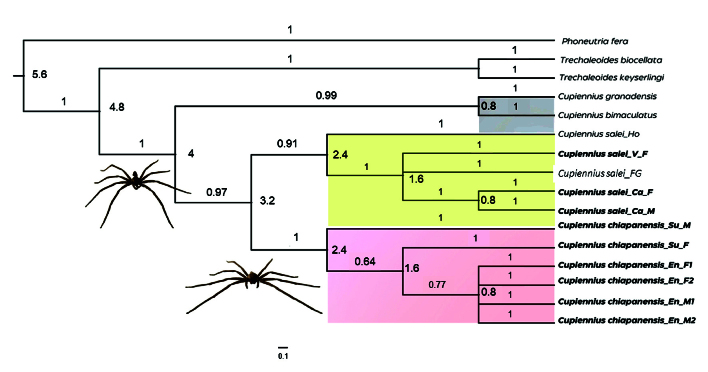
Generation of phylogenetic tree by Bayesian analysis for two *Cupiennius* (Trechaleidae) species. Branch color indicates the difference between species. Clades are identified as *Cupiennius salei* (yellow), *Cupiennius chiapanensis* (red), *Cupiennius bimaculatus,* and *Cupiennius granadensis* (grey). Bootstrap values are indicated on the branches.

The tree shows the genus *Cupiennius* as a monophyletic group, with three monophyletic clades for the included species, indicating a sister relationship between *C. salei* and *C. chiapanensis*. Also, the tree shows that the clade that includes these two genera is the sister group of the clade that joins *C. granadensis* and *C. bimaculatus* as another sister group. The posterior probability and bootstrap values clearly support the separation between these two species (Figure 3). Posterior probability values are indicated (*C. chiapanensis*: 1; *C. salei*: 0.91: *C. granadensis* and *C. bimaculatus*: 0.99) and bootstrap values appear on branches. *Phoneutria fera* was used as an outgroup because it is a wandering spider, like *Cupiennius* spiders, and is commonly found across South American banana plantations [44]. Furthermore, before its formal description as a new species, *C. chiapanensis* had occasionally been mistaken for *P. fera* [7, 45, 46].

A genetic distance analysis (p-distance) was conducted using sequence data from *Cupiennius* species (13 sequences) and the outgroups *Trechaleoides* (2), and *P. fera* (1). A data matrix was generated from 16 sequences and 675 aligned nucleotides from COI sequences. The results show values between 0.3 and 0.4% for *C. chiapanensis* (Additional file 2), and DNA sequence data indicate that specimens from this species from sites 1 (En) and 2 (Su) are related, with nine changes between them (as part of intraspecific variability). The calculated genetic distance also separates *C. chiapanensis* from *C. salei* (average, minimum, and maximum values are 4.3%, 0.33%, and 7.8%, respectively; Additional file 2). A phylogenetic tree was constructed from genetic distance estimates (Additional file 3).

A considerable number of nucleotide changes were detected between *C. salei* sequences from Ca and Ver on the one hand, and from databases on the other (p-distance minimum and maximum values were 3.9% and 7.8%, respectively). The genetic distances between *Cupiennius* spiders and the outgroup taxa are given in Additional file 2. *Cupiennius salei* sequences from Ca and Ver exhibit 96% identity between each other. The alignment of Ca and Ver *C. salei* sequences included data from male as well as female individuals (Additional file 4).

## Haplotype network and genetic diversity

**Figure 4.**
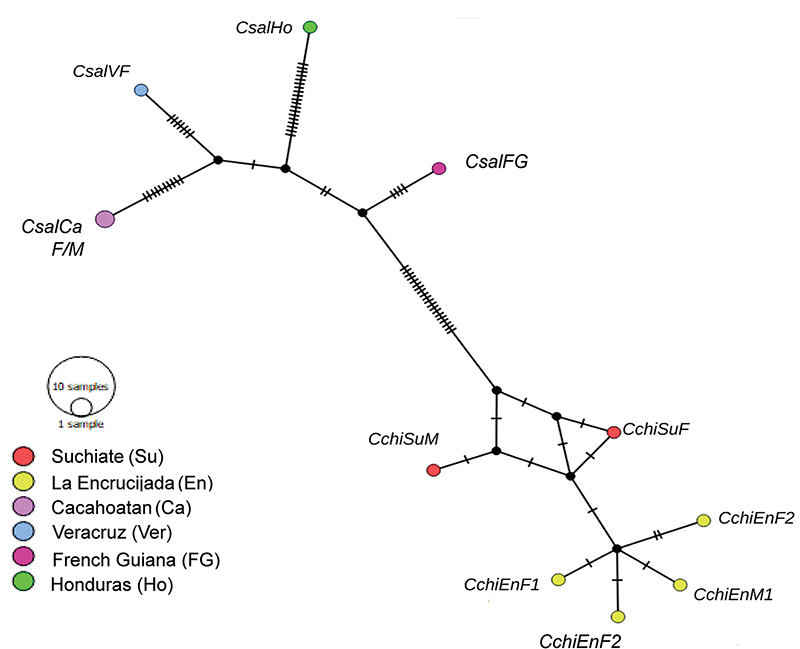
Haplotype network of the wandering spiders *Cupiennius chiapanensis* (below right, *Cchi*) and *Cupiennius salei* (above left, *Csal*).

A haplotype network was created to assess geographic associations among haplotypes using 11 *Cupiennius* COI sequences (452 pb), including database-retrieved *C. salei* sequences from French Guyana and Honduras. The COI sequences were grouped into ten haplotypes (Figure 4), the only common ones being *C. salei* haplotypes from Ca. The haplotype network reveals a geographic association among haplotypes and a separation between *C. chiapanensis* and *C. salei* (20 mutational steps). The *C. chiapanensis* haplotypes from sites En and Su are separated by one mutational step. Likewise, haplotypes from site En are also separated by one mutational step. All sites are separated from each other. The genetic diversity index was computed using the “Compute Variance of Pi” setting, yielding s = 58, h = 10, π = 0.044, K = 20.945, θ = 19.802, and Hd = 0.982. The results agree with species delimitation analyses. Sequences from En and Su belong to the same *C. chiapanensis* haplotype, while those from Ca and Ver belong to the *C. salei* haplotype. However, more than seven intraspecific changes are detected.

## Venomics analysis of *Cupiennius*

**Figure 5.**
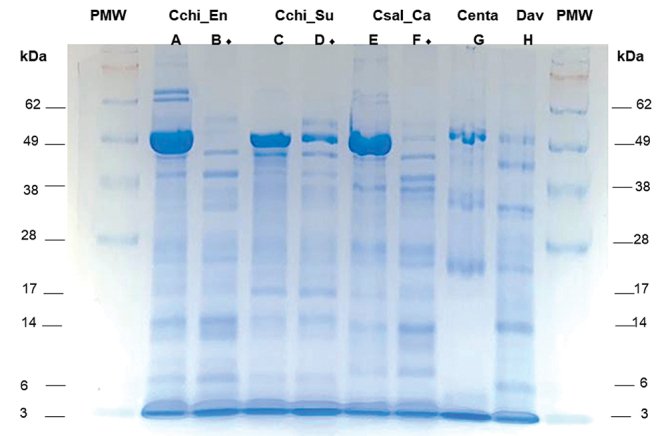
Separation on 12% polyacrylamide gel with sodium dodecyl sulfate (SDS-PAGE). PMW: protein molecular marker, Invitrogen Seeblue Plus2 Pre-stained Protein Standard (Thermo Fisher, USA). **(A, B)** Venom of *Cupiennius chiapanensis* En, Cchi_En (50 µg). **(C, D)** Venom of *Cupiennius chiapanensis* Su, Cchi_Su (50 µg). **(E, F)** Venom of *Cupiennius salei* Ca, Csal_Ca (50 µg). **(G)** Venom of *Centruroides tapachulaensis,* Centa (50 µg). **(H)** Venom sample of *Davus* aff. *pentaloris,* Dav (50 µg). (♦) Milked after having been kept in captivity.

**Figure 6.**
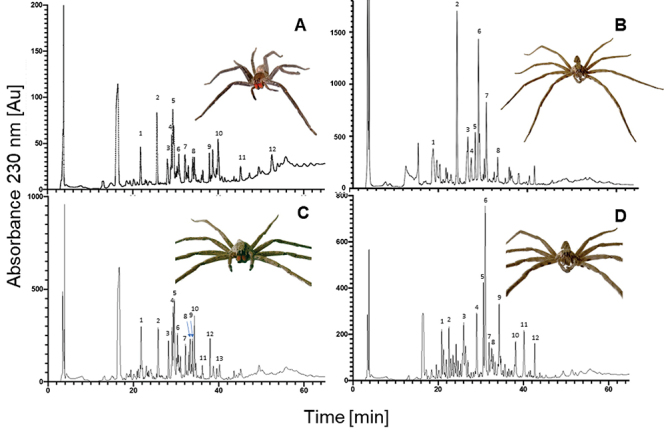
Chromatographic separation of venom from **(A, C)** *Cupiennius chiapanensis* and **(B, D)** *Cupiennius salei* using reversed-phase HPLC. Sites: **(A)** La Encrucijada; **(B)** Cacahoatán; **(C)** Suchiate; **(D)** Veracruz.

**Figure 7.**
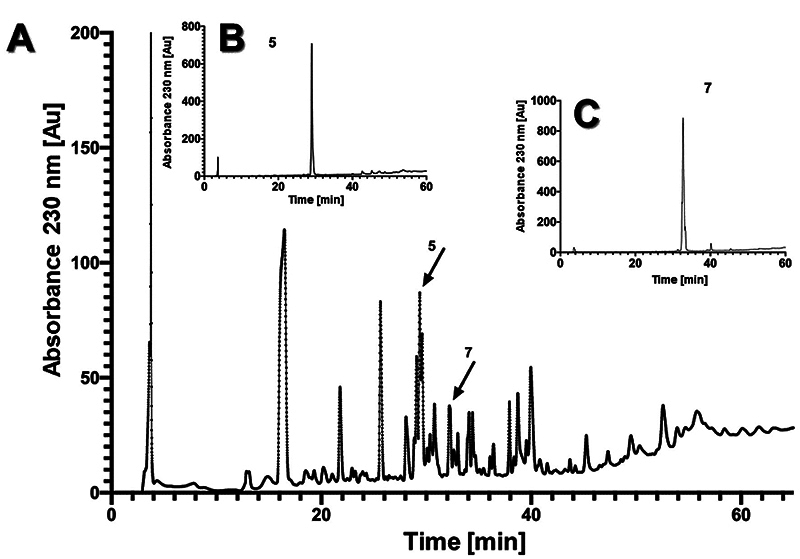
Chromatographic separation of venom using reversed-phase HPLC, and second purification to obtain fractions 5 and 7 of venom from *Cupiennius chiapanensis* from site *La Encrucijada*. **(A)** Venom (1 mg); **(B)** component fraction 5 (ChiaTx1); **(C)** component fraction 7 (ChiaTx2).

Venom was successfully milked from a total of 50 specimens of *C. chiapanensis* and 39 specimens of *C. salei*. After the milking, specimens were fed and kept in captivity for a second milking two -or three- weeks post-collection. The protein profile of *C. chiapanensis* and *C. salei* venom (50 µg) is shown in Figure 5. Several bands with molecular masses ranging from 3 to 62 kDa were detected in the protein profile. Chromatograms of the main venom HPLC fractions are shown in Figure 6. Mass spectrometric analysis of these fractions revealed 23 molecular masses between 585.25 and 7,215.81 Da (Table 2); and two components were sequenced by Edman degradation, (Table 3) for *C. chiapanensis* (Figure 6A and 6C, corresponding to sites En and Su, respectively) and 21 molecular masses between 775.75 and 10,592.00 Da (Table 2, and 4) in *C. salei* (Figures 6 B  and 6D, corresponding to sites Ca and Ver, respectively). Figure 7 shows the chromatographic separation of venom from *C. chiapanensis* collected at site En, and the purification of fractions 5 (ChiaTx1) and 7 (ChiaTx2), which were bio-assayed for toxicity against mammals and insects and subjected to mass spectrometric analysis and sequenced by Edman degradation (Table 3).

**Table 2.** ESI-LC/MS mass fingerprinting (Da) of the venoms of *Cupiennius chiapanensis* (En and Su) and *Cupiennius salei* (Ca and Ver).

La Encrucijada (En)	Suchiate (Su)	Cacahoatán (Ca)	Los Tuxtlas (Ver)
585.25	3809.64	4127.23	775.75
4310.00	4511.86	4237.44	1032.80
4422.50	4623.70	5372.27	1218.15
4534.50	5645.36	5485.87	3740.60
5511.81	5773.19	5510.27	5751.01
5530.50	5774.09	5624.13	5822.57
5752.65	5856.20	5751.37	6285.63
5752.78	5910.45	7275.00	6304.83
6085.26	5928.80	9077.00	6313.01
6166.7*	6170.05	10592.00	6314.42
6268.9*	6271.81		8443.09
	6278.09		
	6320.24		
	7215.81		

Molecular masses in grey correspond to ChiaTx1 (site En), similar mass values are present in both species. Molecular masses obtained from the venom purification fraction 7 from *C. chiapanensis* collected at site En are indicated with an asterisk.

**Table 3.** Alignment and amino acid sequence of ChiaTx1 and ChiaTx2 from *C. chiapanensis* and related sequences.

Toxin name/ ID UniProt	Amino acid sequence	Identity (%)	Exp. mass (Da)	Length (aa)	Activity
CSTX1 | P81694	1 10 20 30 40 50 60 70	100	8352	74	Neurotoxic^a,b^ Ref. [15]
	| | | | | | | |				
	SCIPKHEECTNDKHNCCRKGLFKLKCQCSTFDDESGQPTERCACGRPMGHQAIETGLNIFRGLFKGKKKNKKTK				
**ChiaTx1**	SCIQKHEECTNDRHNCCRKGMFKLKCQCSTFDDE…	88.6	5752	-	Neurotoxic^a^ This work
CSTX-9 | P58604	-DDKNCIPKHHECTNDKKNCCKKGLTKMKCKCFTVADAKGATSERCACDSSLLQKFGFTGLHIIKGLF	100	7530.9	68	Neurotoxic^b^ Ref. [53]
**ChiaTx2**	KDGKNCIPKHHECTNDISNC…	85.0	6166.7 6268.9	-	Neurotoxic^c^ This work

^a^ toxicity against mammals; ^b^ toxicity against insects (flies); ^c^ toxicity against insects (crickets).

**Table 4.** List of experimental and reference (*Cupiennius salei*) molecular masses.

Site	Fraction	Experimental molecular mass (Da) This work	Molecular mass (Da) of putative related protein/toxin	Activity/UniProtKB (access number)	Reference
En	1	UD	-	-	-
En	2	UD	-	-	-
En	3	5511.81	CsTx neurotoxins (between 3700-8300)	Neurotoxic peptides	[14], [48],
En	4	5752.65	CsTx neurotoxins (between 3700-8300)	Neurotoxic peptides	[14],[48]
		5530.50	CsTx neurotoxins (between 3700-8300)	Neurotoxic peptides	
En	5	5752.78	CsTx neurotoxins (between 3700-8300)	Neurotoxic peptides	[14], [48]
En	6	UD	-	-	
En	7	6085.26	CsTx neurotoxins (between 3700-8300)	Neurotoxic peptides	[14], [49]
En	8	4422.50	CsTx neurotoxins (between 3700-8300)	Neurotoxic peptides	[14], [48]
		4534.50	CsTx neurotoxins (between 3700-8300)	Neurotoxic peptides	[14], [49]
		4310.0	CsTx neurotoxins (between 3700-8300)	Neurotoxic peptides	
En	9	UD	-	-	-
En	10	UD	-	-	-
En	11	UD	-	-	-
En	12	585.25	Cupiennin 1 family (998-3800)	Small cationic peptides	[14], [48]
Su	1	UD	-	-	-
Su	2	UD	-	-	-
Su	3	UD	-	-	-
Su	4	5774.09	CsTx neurotoxins (between 3700-8300)	Neurotoxic peptides	[14], [48]
		5856.20	CsTx neurotoxins (between 3700-8300)	Neurotoxic peptides	[14], [50]
Su	5	5773.19	CsTx neurotoxins (between 3700-8300)	Neurotoxic peptides	[14], [49]
		5928.80	CsTx neurotoxins (between 3700-8300)	Neurotoxic peptides	[49]
		5645.36	CsTx neurotoxins (between 3700-8300)	Neurotoxic peptides	[14][14]
Su	6	3809.64	Cupiennin 1 family (998-3800)	Small cationic peptides; Cupiennin-1b (P83620)	[14], [49]
		5910.45	CsTx neurotoxins (between 3700-8300)	Neurotoxic peptides	[14]
Su	7	7215.81	CsTx neurotoxins (between 3700-8300)	Neurotoxic peptides	[14], [49]
Su	8	6278.09	CsTx neurotoxins (between 3700-8300)	Neurotoxic peptides	[14]
Su	9	6320.24	CsTx neurotoxins (between 3700-8300)	Neurotoxic peptides	[14]
Su	10	6271.81	CsTx neurotoxins (between 3700-8300)	Neurotoxic peptides	[14]
Su	11	4511.86	CsTx neurotoxins (between 3700-8300)	Neurotoxic peptides	[48]
		4623.70	CsTx neurotoxins (between 3700-8300)	Neurotoxic peptides	[49]
Su	12	6170.05	CsTx neurotoxins (between 3700-8300)	Neurotoxic peptides	[14]
Su	13	UD	-	-	-
Ca	1	UD	-	-	-
Ca	2	UD	-	-	-
Ca	3	5510.27	CsTx neurotoxins (between 3700-8300);	Neurotoxic peptides	[14], [48]
			Protein Krueppel (5546.0)	Regulatory protein	[49]
		5372.27	CsTx neurotoxins (between 3700-8300)	Neurotoxic peptides	
Ca	4	9077.0	CsTx peptides with colipase MIT1-like fold (between 7200-9901)	The acidic peptides (CsTx20 and CsTx21)	[14], [48]
		10592.0	Enzymes and proteins (above 10000)		[14], [48]
		7275.0	CsTx neurotoxins (between 3700-8300)	Neurotoxic peptides	[14], [48]
Ca	5	5624.13	CsTx neurotoxins (between 3700-8300)	Neurotoxic peptides	[14], [48]
Ca	6	5751.37	CsTx neurotoxins (between 3700-8300)	Neurotoxic peptides	[14], [48]
Ca	7	5485.87	CsTx neurotoxins (between 3700-8300)	Neurotoxic peptides	[50]
Ca	8	4127.23	CsTx neurotoxins (between 3700-8300)	Neurotoxic peptides	
		4237.44	CsTx neurotoxins (between 3700-8300)	Neurotoxic peptides	[14], [48]
			CsTx neurotoxins (between 3700-8300)	Neurotoxic peptides	[14], [48]
Ver	1	1032.80	Cupiennin family (998-3800)	Small cationic peptides; toxin activity	
Ver	2	1218.15	Cupiennin family (998-3800)	Small cationic peptides; toxin activity	
Ver	3	3740.60	Cupiennin family (998-3800); CsTx neurotoxins (between 3700-8300)	Small cationic peptides; antimicrobial neurotoxin	[14], [48], [51]
Ver	4	5751.01	CsTx neurotoxins (between 3700-8300)	Neurotoxic peptides	[14], [48]
Ver	5	6285.63	CsTx neurotoxins (between 3700-8300)	Neurotoxic peptides	[49]
		6313.01	CsTx neurotoxins (between 3700-8300)	Neurotoxic peptides	[49]
Ver	6	5822.57	CsTx neurotoxins (between 3700-8300)	Neurotoxic peptides	[49]
Ver	7	6314.42	CsTx neurotoxins (between 3700-8300)	Neurotoxic peptides	[49]
Ver	8	775.75	Cupiennin family (998-3800)	Small cationic peptides; toxin activity	[48]
Ver	9	6304.83	CsTx neurotoxins (between 3700-8300)	Neurotoxic peptides	[48]
Ver	10	UD	-	-	-
Ver	11	8443.09	CsTx neurotoxins (between 3700-8300)	Neurotoxic peptides	[11]
Ver	12	UD	-	-	

UD: undetermined; - represent no data.

Table 4 compares experimental mass values from all four collection sites with nearby mass values retrieved from the UniProtKB database, and includes reference values retrieved for *C. salei* and additional peptide information related to post-translational modifications [52, 53].

The amino acid sequences of ChiaTx1 (fraction 5; 5,752 Da; Figure 6 A  and 7B) and ChiaTx2 (fraction 7, 6268.9, and 6166.7 Da; Figure 7 C) from *C. chiapanensis* show similarity to toxins previously reported by Kuhn-Nentwig et al. [51] and Kuhn-Nentwig et al. [16].

## Biological effects of *Cupiennius* venom and venom fractions

The toxic effects of venom were studied in *Anastrepha obliqua.* Injected flies were observed for signs of paralysis of wings and legs, contractions, spasms, or complete paralysis (Table 5). Toxicity became apparent at 20 μg; at higher doses (40 and 50 μg of venom), death occurred after 30 minutes. Venom from *C. tapachulaensis* was used as insect toxicity control. At a dose of 40 and 50 μg, venom from *C. chiapanensis* induced paralysis after five minutes, followed by death after 30-40 minutes (Table 6).

The purified fractions 5 (ChiaTx1) and 7 (ChiaTx2) were bio-assayed for toxicity. ChiaTx1 produced signs of toxicity in mice at 1 μg/mouse (bristly hair, ptosis, ataxia), while ChiaTx2 induced paralysis in crickets at 1.2 μg/cricket. 

**Table 5.** Symptoms observed in *Anastrepha obliqua* flies after injection with venom from *Cupiennius chiapanensis, Cupiennius salei,* and the scorpion *Centruroides tapachulaensis.*

Symptoms observed in prey model			
Symptoms	Venom		
	*C. chiapanensis*	*C. salei*	*Cen. tapachulaensis*
Inability to move wings	+	++	+++
Loss of leg movement	++	+++	++
Paralysis	+++	+++	+++

Symbols describe the intensity of the symptoms: +, low; ++, moderate; +++ high intensity. Trials consisted of five flies that were injected in the central tergite (20 μg).

**Table 6.** Toxic activity of venom from *Cupiennius chiapanensis, Cupiennius salei,* and *Centruroides tapachulaensis.*

Venom	Conc. (μg/ μL)	Effect in flies		Prey mass (mg)	Reaction time (min)	Lethal time (min)	Number of flies
		*Paralytic*	*Lethal*				
*C. chiapanensis*	50	++	+++	14.76	3	30	5
*C. salei*	50	++	+++	17.2	0	20	5
*Cen. tapachulaensis*	50	+++	+++	15.32	0	50	5
*Cen. chiapanensis*	40	++	+++	14.73	5	40	10
*C. salei*	40	+++	+++	14.25	0	30	10
*C. chiapanensis*	20	++	+++	14.94	5	60	10
*C. salei*	20	+++	+++	14.58	1	60	10
*Cen. tapachulaensis*	20	+++	+++	13.66	1	60	5

Symbols describe the intensity of the symptoms: +, low; ++, moderate; +++ high intensity. This table shows the post-injection time required for toxic effects to manifest. Lethality was observed in the fly *Anastrepha obliqua*.

## Discussion

Our results show that the distribution of *C. chiapanensis* is not limited to *La Encrucijada*, the Biosphere reserve from where it was first described in 2006. This species has been observed in two different ecosystems - mangrove and agricultural land - and has been found in different vegetation types. The mangrove and palm grove ecosystems that typify *La Encrucijada* (En) are located 76 km from agricultural Suchiate (Su), showing that this species has managed to adapt to an ecosystem disturbed by the introduction of banana monoculture, as was observed for *C. salei* in Central America as well, and to adhere to certain habitats [6, 51]. *Cupiennius salei* arrived in Europe on shipments of bananas and became the subject of active scientific investigation there in the 1960s [6, 14]. 

In the state of Chiapas, banana is an important economic crop, and the need for new agricultural land has led to a reduction of the original vegetation. The state of Chiapas, where three of the sampling sites are located, produces 696,000 tons of bananas annually [54] and *C. chiapanensis* has been observed at the banana plantation collection site. What the data do seem to indicate so far is that, at least in Mexico, the distribution of *C. chiapanensis* is restricted to low altitudes (En and Su are at 26 and 33 m a.s.l., respectively), while *C. salei* is often found at higher altitudes (Ca and Ver are at 665 and 150 m a.s.l., respectively). In 2002 Barth recorded *C. salei* from high-altitude localities in Mexico (Fortín de las Flores, 1006 m a.s.l.) and Guatemala (Finca Remedios, 700 m a.s.l.) [6], whereas Medina [7] recorded *C. salei* from the *La Encrucijada* Biosphere reserve (sea level) only during the rainy season.

*Cupiennius salei* was previously recorded from the state of Veracruz (Fortín de las Flores) [6], and in the present study, a female specimen of this species was collected in Ver, which is located at a different site but in the same state. Site Ver is located more than 400 km away from Ca. Taxonomical identification and COI sequence data coincided in that specimens collected from Ca and Ver belong to *C. salei*. Sequence alignments revealed the presence of more than 20 changes (Additional file 4). Species delimitation, moreover, was determined by sequence analysis for the four collection sites. The average genetic distance - a measure of divergence [47] - between *C. chiapanensis* and *C. salei* was 4.3%. The average intraspecific genetic distance for *C. chiapanensis* was less than 1%, indicating that the sequences belonged to the same species [55, 56]). However, the sampling and analysis of individuals allowed us to compare a species of restricted distribution with other species from the same population, and to recognize an intraspecific variation between sexes (Additional file 4).

The inferred phylogenetic tree from Figure 2 shows the relationship and separation between the clades of *C. chiapanensis*, *C. salei*, a group of other *Cupiennius* spiders (*C. granadensis* and *C. bimaculatus*), and two spiders from the genus *Trechaleoides* (which, like *Cupiennius*, belongs to the family Trechaleidae). Sequences obtained from *C. salei* exhibited greater variability and changes in the COI region in comparison with those from *C. chiapanensis*. This observation is confirmed by the haplotype network, which shows 20 mutational steps between their respective haplotypes (Figure 3). *Cupiennius salei* reportedly has a wider distribution, which might indicate that this species possesses the ability to adapt to diverse habitats. Therefore, it is important to collect specimens from unexplored regions in Central America, so that its COI variability can be mapped more precisely. *Cupiennius chiapanensis*, on the other hand, is characterized by a more limited distribution. So far, this species has not been reported from outside of Chiapas, which could be explained by its apparent restriction to lower altitudes (like the Chiapas coastal region) or, as is the case for *C. salei*, its preference for large-leaved monocots as shelter sites [6]. This feature, however, has allowed it to occupy areas where land use has recently been changed, such as the banana agricultural zone at Su.

A mass fingerprint was generated for *C. chiapanensis* as well as *C. salei*, and their venom components were compared. The chromatographic venom profiles were different between species, but similar between the collection sites of *C. chiapanensis*. Interestingly, the venom of both species contained a compound with molecular mass 5,751 - 5,752 Da, designated ChiaTx1 in the present study (Table 2, sites En, Ca, and Ver). This indicates that it could be a conserved peptide, turning it into an intriguing target for future investigations. Owing to the limited amount of available samples, the toxicity of ChiaTx1 could only be confirmed in mice. The values of the other molecular masses were similar to values for toxins reported from *C. salei* (for instance 5,773.19 Da, 5,774.09 Da, and 5,928.8 Da), so these components might be related to peptides with toxicity against insects. The sequence of the peptide ChiaTx2 shows 85% identity with the first 20 residues of CSTX-9 from *C. salei* (a peptide that is toxic to flies). ChiaTx2 (6,268.9 and 6,166.7 Da) was bio-assayed for toxicity against crickets and observed to induce paralysis. Our study is the first to provide bio-assay data for ChiaTx1 and ChiaTx2. 

The complexity of spider venom is not only understood in terms of the number and activity of its components, but also in terms of the synergistic interactions between these components that maximize their potency [11]. For the venom of *C. salei*, synergy refers to the primary function of some peptides to enhance the bioactivity of others. The toxicity of CSTX-1, for example, is increased in the presence of CSTX-13 [57]. The ChiaTx1 peptide shows 88% identity with CSTX-1 (in the 34 amino acid N-terminal region, Table 3). They have different molecular masses, however, and our data indicate that ChiaTx1 is smaller than CSTX-1.

Venom from *C. salei* has been extensively studied [11, 14]. Several protein and peptide families have been described, the cupiennins (Cu) being one of them (mass range 998.0 - 3,800 Da) [14]. Cu are lysine-rich cationic peptides with a molecular weight of 1 - 4 kDa that are characterized by their cytolytic activity [12]. Our results contain molecular masses related to cupiennin peptides. Cu1 and Cu2 families adopt an α-helical structure, which confers strong cytolytic activity to them [51]. Cupiennin1a (3796.17 Da) was reported to increase the activity of the toxins CSTX-1 and CSTX-9 up to 65% [14]. In the present study, we found mass values within the cupiennin range, for example, Cu1b (3,800.25 Da) and Cu1c (3,769.75 Da) [42], in venom from both species (Table 2, sites Su and Ver). Tests in *Drosophila melanogaster* showed that these cupiennins possessed insecticidal activity [16, 41]. Also, we detected molecular masses that are related to neurotoxic compounds, cupiennins, and small linear cationic peptides (SCP), which previously were reported by Kuhn-Nentwig et al. [14] (Table 4). 

The present study advances our understanding of the venom composition of *Cupiennius* spiders. The molecular masses that we detected in venom from *C. salei* did not perfectly match values from databases, although these differences may be due to the use of different analysis methods [48]. The use of multiple MS methodologies for the analysis of spider venom can provide complementary information for the generation of a complete mass fingerprint [11, 58]. In the present investigation, mass spectrometric data were generated using ESI-MS; other studies have characterized *C. salei* venom using MALDI-TOF-MS, LC-MS, and ESI-MS [11, 48, 49]. 

The amino acid sequence of ChiaTx1 (Table 3) shows 88% identity with CSTX-1 (the first 35 amino acids of the sequence), a toxin with a length of 74 amino acids (GenBank Accession: AAB31115.1) that contains an ICK motif at the *N*-terminus and possesses cytolytic activity at its α-helical *C*-terminus [13, 52]. This neurotoxin blocks L-type calcium channels (CaV1/CACNA1) in mammalian neurons at nanomolar levels [59]. We bioassayed ChiaTx1 for activity against mammals and observed a toxic effect in mice (bristly hair, slow movement). A transcriptome analysis of *C. salei* by Kuhn-Nentwig et al. [11], revealed the presence of several gene families that encode precursor sequences, including a signal peptide and peptides with an ICK motif and α-helical C-terminus (family SN_19). ChiaTx1 shows higher similarity to members of the SN_19_3 family (CsTx-1a,b,c, CsTx-10a,b) [11]. The molecular mass of ChiaTx1 was detected in venom fractions from *C. chiapanensis* as well as *C. salei*, and is perhaps a constituent of the toxin arsenal these spiders have at their disposal for capturing and subduing prey. ChiaTx2 (Table 3) showed 85% similarity to the CSTX-9 toxin (first 20 residues), which is toxic to insects (*Drosophila melanogaster*, LD_50_ = 3.12 pmol/mg) [11, 60]. Similar to CSTX-9, ChiaTx2 was observed to induce paralysis in crickets at 1.2 μg/cricket.

Our investigation generated the first DNA barcoding sequences (using COI) of *C. chiapanensis* and presented the first characterization of its venom. Spider venoms are a rich and diverse source of unique compounds, some of which are affected by natural habitat, feeding behavior, and abiotic factors [49]. We observed differences between the chromatographic profiles and molecular mass values from both species. Also, we detected broad bands of high-molecular-weight compounds (> 45 kDa) in venom from freshly collected specimens, but not in that from individuals in captivity. We currently lack the necessary data to explain this observation.

Our investigation explored different ecosystems and contributed data that will enable us to gain new insight into the distribution of *Cupiennius* spiders. *Cupiennius salei* searches for monocotyledonous plants - such as *Musa* sp, and *Aechmea* sp - that shelter them. During our fieldwork, we observed *C. chiapanensis* seeking shelter in the leaf base of palms *Sabal* sp, not formally identified but likely *Sabal mexicana*; where palm leaves are attached to the trunk [7], which are part of the native mangrove vegetation, and below the sheaths of outer leaves of banana pseudostems. Our study also provides new data that can be used for the development of conservation strategies for this species. Moreover, given the encouraging bioassay results for venom from both species, exploration of their biotechnological and biomedical potential can foster the development of new applications.

## Conclusion

The present study is the first to report on the analysis of venom from *Cupiennius* spiders from southern Mexico. It focuses on two species collected from Chiapas - *C. salei* and endemic *C. chiapanensis* - that were identified and characterized by DNA barcoding analysis using the COI gene. This enabled us to infer a phylogenetic tree and study its relationship to other species from the same genus from the Americas. Chromatographic and mass spectrometric data allowed us to identify two new toxins from the genus *Cupiennius*. Our data provide new insights into the distribution, haplotypes, and venom components of these species, and open the door to the further exploration of their biotechnological and biomedical potential.

## Abbreviations

BIC: Bayesian information criterion; COI: cytochrome c oxidase subunit I; CSTX: toxin from *Cupiennius salei*; Cu: cupiennins; ChiaTx: toxin from *Cupiennius chiapanensis;* En: Site 1 (*La Encrucijada*); Su: Site 2 (Suchiate); Ca: Site 3 (Cacahoatán); Ver: Site 4 (Veracruz); PCR: polymerase chain reaction; GTR: The General Time Reversible; MCMC: Markov Chain Monte Carlo; TFA: trifluoroacetic acid; ESI-MS: electrospray ionization mass spectrometry; HPLC: high-performance liquid chromatography.

## Availability of data and materials

 The data supporting the findings of this study are available from the corresponding author EDG on request.

## Supplementary material

The following online material is available for this article:

Additional file 1. List of collected and identified *Cupiennius* spider specimens.

Additional file 2. Matrix of genetic distances (p-distance) between *Cupiennius chiapanensis* and *Cupiennius salei* from southern Mexico.

Additional file 3. Matrix for the genus *Cupiennius*.

Additional file 4. Multiple sequence alignments of COI gene of *Cupiennius salei* and *Cupiennius chiapanensis.*
