# Supplementary material for: Cupiennius spiders (Trechaleidae) from southern Mexico: DNA barcoding, venomics, and biological effect
Source: J Venom Anim Toxins Incl Trop Dis. 2024 Aug 12;30:e20230098. doi: 10.1590/1678-9199-JVATITD-2023-0098 (PMC11333084; doi:10.1590/1678-9199-JVATITD-2023-0098)
Supplement: Additional file 1. [file 1678-9199-jvatitd-30-e20230098-s1.pdf]

J Venom Anim Toxins incl Trop Dis,
https://doi.org/10.1590/1678-9199-JVATITD-2023-0098

Supplementary Material to "*Cupiennius* spiders (Trechaleidae) from southern Mexico: DNA barcoding, venomics, and biological effect"

Additional file 1. List of collected and identified *Cupiennius* spider specimens.

List of collected specimens								
**ID**	**Male**	**Pre-male**	**Female**	**Pre-female**	**Juvenile**	**Total**	**Locality**	**Coordinates**
Cupiennius salei	5	2	9	2	19	37	Cacahoatán	15°02'18.3"N 92°10'18.6"W
Cupiennius salei	0	0	0	0	1	1	Cacahoatán	15°09'28.5"N 92°18'13.6"W
Cupiennius salei	0	1	2	0	0	3	Reserva de Los Tuxtlas	18°35' 04" N 95°04' 26" W
Cupiennius chiapanensis	3	4	2	7	14	30	Suchiate	14°38' 39" N 92°11' 52" W
Cupiennius chiapanensis	0	2	3	7	8	20	Acapetahua	15°12' 37" N 92°53' 58.3" W
