# Supplementary material for: Cupiennius spiders (Trechaleidae) from southern Mexico: DNA barcoding, venomics, and biological effect
Source: J Venom Anim Toxins Incl Trop Dis. 2024 Aug 12;30:e20230098. doi: 10.1590/1678-9199-JVATITD-2023-0098 (PMC11333084; doi:10.1590/1678-9199-JVATITD-2023-0098)
Supplement: Additional file 2. [file 1678-9199-jvatitd-30-e20230098-s2.pdf]

J Venom Anim Toxins incl Trop Dis,
https://doi.org/10.1590/1678-9199-JVATITD-2023-0098

Supplementary Material to "*Cupiennius* spiders (Trechaleidae) from southern Mexico: DNA barcoding, venomics, and biological effect"

Additional file 2. Matrix of genetic distances (p-distance) between *Cupiennius chiapanensis* and *Cupiennius salei* from southern Mexico.

	1	2	3	4	5	6	7	8	9	10	11
1. Cchi_Su_M											
2. Cchi_Su_F	0.0033										
3. Cchi_En_F 1	0.0067	0.0033									
4. Cchi_En_F 2	0.0137	0.0102	0.0067								
5. Cchi_En_M 1	0.0102	0.0067	0.0033	0.0102							
6. Cchi_En_M 2	0.0101	0.0067	0.0033	0.0101	0.0067						
7. Csal_CaF	0.0619	0.0579	0.0619	0.0702	0.0579	0.0657					
8. Csal_CaM	0.0619	0.0579	0.0619	0.0702	0.0579	0.0657	0.0000				
9. Csal_VF	0.0699	0.0657	0.0699	0.0783	0.0741	0.0738	0.0465	0.0465			
10. Csal_GF	0.0501	0.0463	0.0501	0.0581	0.0541	0.0539	0.0430	0.0430	0.0390		
11. Csal_Ho	0.0463	0.0424	0.0463	0.0541	0.0501	0.0500	0.0497	0.0497	0.0496	0.0460	

Cchi: *Cupiennius chiapanensis*; Csal: *Cupiennius salei*; En: Site - 1 La Encrucijada; Su: Site - 2 Suchiata; Ca: Site - 3 Cacahoatán; Ver: Site - 4 Los Tuxtlas; M: male; F: female.
