# Supplementary material for: Cupiennius spiders (Trechaleidae) from southern Mexico: DNA barcoding, venomics, and biological effect
Source: J Venom Anim Toxins Incl Trop Dis. 2024 Aug 12;30:e20230098. doi: 10.1590/1678-9199-JVATITD-2023-0098 (PMC11333084; doi:10.1590/1678-9199-JVATITD-2023-0098)
Supplement: Additional file 3. [file 1678-9199-jvatitd-30-e20230098-s3.pdf]

J Venom Anim Toxins incl Trop Dis,
https://doi.org/10.1590/1678-9199-JVATITD-2023-0098

Supplementary Material to "*Cupiennius* spiders (Trechaleidae) from southern Mexico: DNA barcoding, venomics, and biological effect"

Additional file 3. Matrix for the genus *Cupiennius*.

	1	2	3	4	5	6	7	8	9	10	11	12	13	14	15	16
1. Cchi_Su_M																
2. Cchi_Su_F	0.003															
3. Cchi_En_F1	0.007	0.003														
4. Cchi_En_F2	0.014	0.010	0.007													
5. Cchi_En_M1	0.010	0.007	0.003	0.010												
6. Cchi_En_M2	0.010	0.007	0.003	0.010	0.007											
7. Csal_CaF	0.062	0.058	0.062	0.070	0.058	0.066										
8. Csal_CaM	0.062	0.058	0.062	0.070	0.058	0.066	0.000									
9. Csal_VF	0.070	0.066	0.070	0.078	0.074	0.074	0.046	0.046								
10. Cgranadensis	0.074	0.070	0.074	0.083	0.079	0.078	0.086	0.086	0.090							
11. Csal_GF	0.050	0.046	0.050	0.058	0.054	0.054	0.043	0.043	0.039	0.082						
12. Csal_Ho	0.046	0.042	0.046	0.054	0.050	0.050	0.050	0.050	0.050	0.070	0.046					
13. Cbimaculatus	0.058	0.054	0.058	0.066	0.062	0.062	0.078	0.078	0.073	0.036	0.057	0.062				
14. Phoneutria_fera	0.187	0.182	0.187	0.187	0.192	0.187	0.182	0.182	0.152	0.198	0.167	0.167	0.177			
15. Trechaleoides_biocellata	0.129	0.124	0.129	0.138	0.133	0.133	0.148	0.148	0.152	0.139	0.138	0.115	0.125	0.183		
16. Trechaleoides_keyserlingi	0.129	0.124	0.129	0.138	0.134	0.133	0.143	0.143	0.133	0.135	0.129	0.111	0.102	0.167	0.092	

Cchi: *Cupiennius chiapanensis*; Csal: *Cupiennius salei*; En: Site - 1 La Encrucijada; Su: Site - 2 Suchiata; Ca: Site - 3 Cacahoatán; Ver: Site - 4 Los Tuxtlas; M: male; F: female.
