# Supplementary material for: Cupiennius spiders (Trechaleidae) from southern Mexico: DNA barcoding, venomics, and biological effect
Source: J Venom Anim Toxins Incl Trop Dis. 2024 Aug 12;30:e20230098. doi: 10.1590/1678-9199-JVATITD-2023-0098 (PMC11333084; doi:10.1590/1678-9199-JVATITD-2023-0098)
Supplement: Additional file 4. [file 1678-9199-jvatitd-30-e20230098-s4.pdf]

J Venom Anim Toxins incl Trop Dis,

https://doi.org/10.1590/1678-9199-JVATITD-2023-0098

Supplementary Material to "*Cupiennius* spiders (Trechaleidae) from southern Mexico: DNA barcoding, venomics, and biological effect"

Additional file 4. Multiple sequence alignments of COI gene of *Cupiennius salei* and *Cupiennius chiapanensis*.

```
GenBank|ID                Sequence                                      nt

OR906093|Csal|Ca_F   TATAAGAGTATTAATTCGTATAGAATTAGGACATTCTGGAAGATTATTAGGAGATGATCA   60
OR906095|Csal|V_F    TATAAGAGTATTAATTCGGATAGAATTAGGACATTCTGGAAGATTGTTAGGGGATGATCA   60
KM225104.1|Csal|GF   TATAAGAGTATTAATTCGGATAGAATTAGGACATTCCGGAAGATTATTAGGAGATGATCA   60
ACG3675|Csal|Ho      TATAAGAGTGTTGATTCGAATAGAATTAGGTCATTCTGGAAGTTTATTAGGAGATGATCA   60
OR906087|Cchi|Su_M   TATAAGAGTATTGATTCGAATGGAATTAGGACATTCTGGAAGATTATTAGGGGATGATCA   60
OR906088|Cchi|Su_F   TATAAGAGTATTGATTCGAATGGAATTAGGACATTCTGGAAGATTATTAGGGGATGATCA   60
OR906090|Cchi|En_F2  TATAAGAGTATTGATTCGAATGGAATTAGGACATTCTGGAAGATTATTAGGGGATGATCA   60
OR906089|Cchi|En_F1  TATAAGGGTATTGATTCGAATGGAATTAGGACATTCTGGAAGATTATTAGGGGATGATCA   60
OR906091|Cchi|En_M1  TATAAGAGTATTGATTCGAATGGAATTAGGACATTCTGGAAGATTATTAGGGGATGATCA   60
OR906092|Cchi|En_M2  TATAAGAGTATTGATTCGAATGGAATTAGGACATTCTGGAAGATTATTAGGGGATGATCA   60

OR906093|Csal|Ca_F   TTTATATAATGTAATAGTAACTGCTCATGCATTTGTTATAATTTTTTTTATAGTTATACC 120
OR906095|Csal|V_F    TTTATATAATGTAATAGTGACTGCTCATGCTTTTGTTATAATTTTTTTTATAGTTATACC 120
KM225104.1|Csal|GF   TTTATATAATGTAATAGTAACCGCTCACGCTTTTGTTATAATTTTTTTTATAGTTATACC 120
ACG3675|Csal|Ho      TTTATATAATGTGATAGTTACTGCTCATGCTTTTATTATAATTTTTTTTATAGTTATACC 120
OR906087|Cchi|Su_M   TTTATATAATGTGATGGTGACTGCTCATGCTTTTGTTATAATTTTTTTTATGGTTATACC 120
OR906088|Cchi|Su_F   TTTATATAATGTGATGGTGACTGCTCATGCTTTTGTTATGATTTTTTTTATGGTTATACC 120
OR906090|Cchi|En_F2  TTTATATAATGTGATGGTGACTGCTCATGCTTTTGTTATGATTTTTTTTATGGTTATACC 120
OR906089|Cchi|En_F1  TTTATATAATGTGATGGTGACTGCTCATGCTTTTGTTATGATTTTTTTTATGGTTATATC 120
OR906091|Cchi|En_M1  TTTATATAATGTGATGGTGACTGCTCATGCTTTTGTTATGATTTTTTTTATGGTTATACC 120
OR906092|Cchi|En_M2  TTTATATAATGTGATGGTGACTGCTCATGCTTTTGTTATGATTTTTTTTATGGTTATACC 120

OR906093|Csal|Ca_F   TATTTTAATTGGGGGTTTTGGTAATTGGTTAGTTCCTTTAATATTAGGTGCTCCTGATAT 180
OR906095|Csal|V_F    TATTTTAATTGGAGGTTTTGGTAATTGATTAGTTCCTTTAATATTAGGTGCTCCTGATAT 180
KM225104.1|Csal|GF   TATTTTAATTGGAGGTTTTGGTAATTGATTAGTTCCTTTAATATTAGGTGCTCCTGATAT 180
ACG3675|Csal|Ho      TATTTTAATTGGAGGCTTTGGTAATTGGTTAATTCCTTTGATGTTAGGTGCCCCTGATAT 180
OR906087|Cchi|Su_M   TATTTTAATTGGTGGTTTTGGAAATTGATTGGTTCCTTTAATATTAGGTGCTCCTGATAT 180
OR906088|Cchi|Su_F   TATTTTAATTGGTGGTTTTGGAAATTGATTGGTTCCTTTAATATTAGGTGCTCCTGATAT 180
OR906090|Cchi|En_F2  TATTTTAATTGGTGGTTTTGGAAATTGATTGGTTCCTTTAATATTAGGTGCTCCTGATAT 180
OR906089|Cchi|En_F1  TATTTTAATTGGTGGTTTTGGAAATTGATTGGTTCCTTTAATATTAGGTGCTCCTGATAT 180
OR906091|Cchi|En_M1  TATTTTAATTGGTGGTTTTGGAAATTGATTGGTTCCTTTAATATTAGGTGCTCCTGATAT 180
OR906092|Cchi|En_M2  TATTTTAATTGGTGGTTTTGGAAATTGATTGGTTCCTTTAATATTAGGTGCTCCTGATAT 180

OR906093|Csal|Ca_F   ATCATTTCCTCGAATAAATAATTTGTCTTTTTGATTACTTCCTCCTTCTTTATTTTTGTT240
OR906095|Csal|V_F    ATCATTTCCTCGGATAAATAATTTGTCTTTTTGATTACTTCCTCCTTCTTTATTTTTGTT 240
KM225104.1|Csal|GF   ATCATTTCCTCGAATAAATAATTTGTCTTTTTGATTACTTCCCCCTTCTTTATTTTTATT 240
ACG3675|Csal|Ho      ATCATTTCCTCGTATAAATAATTTGTCTTTTTGATTATTACCTCCTTCTTTATTTTTATT 240
OR906087|Cchi|Su_M   GTCATTTCCTCGGATAAATAATTTATCTTTTTGATTATTACCTCCTTCTTTGTTTTTATT 240
OR906088|Cchi|Su_F   GTCATTTCCTCGTATAAATAATTTATCTTTTTGATTATTACCTCCTTCTTTGTTTTTATT 240
OR906090|Cchi|En_F2  GTCATTTCCTCGGATAAATAATTTATCTTTTTGATTATTACCTCCTTCTTTGTTTTTATT 240
OR906089|Cchi|En_F1  GTCATTTCCTCGGATAAATAATTTATCTTTTTGATTATTACCTCCTTCTTTGTTTTTATT 240
OR906091|Cchi|En_M1  GTCATTTCCTCGGATAAATAATTTATCTTTTTGATTATTACCTCCTTCTTTGTTTTTATT 240
OR906092|Cchi|En_M2  GTCATTTCCTCGGATAAATAATTTATCTTTTTGATTATTACCTCCTTCTTTGTTTTTATT 240

OR906093|Csal|Ca_F   ATTTATATCATCAATAGTAGAAATAGGTGTGGGAGCAGGATGAACAGTTTATCCTCCTTT 300
OR906095|Csal|V_F    ATTTATATCATCGATAGTAGAAATAGGTGTAGGAGCGGGATGAACTGTTTATCCTCCTTT 300
KM225104.1|Csal|GF   ATTTATATCATCGATAGTGGAAATAGGTGTAGGAGCAGGATGGACTGTTTATCCTCCTTT 300
ACG3675|Csal|Ho      ATTTATATCATCTATAGTAGAAATAGGTGTAGGAGCAGGATGAACTGTTTATCCTCCTTT 300
OR906087|Cchi|Su_M   ATTTATATCATCTATAGTGGAAATAGGTGTGGGAGCAGGATGAACTGTGTATCCTCCTTT 300
OR906088|Cchi|Su_F   ATTTATATCATCTATAGTGGAAATAGGTGTGGGAGCAGGATGAACTGTGTATCCTCCTTT 300
OR906090|Cchi|En_F2  ATTTATATCATCTATAGTGGAAATAGGTGTGGGAGCAGGATGAACTGTGTATCCTCCTTT 300
OR906089|Cchi|En_F1  ATTTATATCATCTATAGTGGAAATAGGTGTGGGAGCAGGATGAACTGTGTATCCTCCTTT 300
OR906091|Cchi|En_M1  ATTTATATCATCTATAGTGGAAATAGGTGTGGGAGCAGGATGAACTGTGTATCCTCCTTT 300
OR906092|Cchi|En_M2  ATTTATATCATCTATAGTGGAAATAGGTGTGGGAGCAGGATGAACTGTGTATCCTCCTTT 300
```

```
OR906093|Csal|Ca_F  AGCTTCTAGAGTTGGTCATATAGGAAGTTCTATGGATTTTGCTATTTTTCTCTTCATTT 360
OR906095|Csal|V_F   AGCTTCTAGAGTTGGTCATATAGGAAGTTCTATAGATTTTGCTATTTTTCTCTTCATTT 360
KM225104.1|Csal|GF  AGCTTCTAGAGTTGGTCATATAGGAAGTTCTATAGATTTTGCTATTTTTCTCTTCATTT 360
ACG3675|Csal|Ho     AGCTTCAAGAGTTGGGCATATAGGAAGTTCTATAGATTTTGCTATTTTTCTCTTCATTT 360
OR906087|Cchi|Su_M  AGCTTCTAGAGTTGGTCATATAGGAAGTTCTATAGATTTTGCTATTTTTCTCTTCATTT 360
OR906088|Cchi|Su_F  AGCTTCTAGAGTTGGTCATATAGGAAGTTCTATAGATTTTGCTATTTTTCTCTTCATTT 360
OR906090|Cchi|En_F2 GGCTTCTAGAGTTGGTCATATAGGAAGTTCTATAGATTTTGCTATTCTTTCTCTTCATTT 360
OR906089|Cchi|En_F1 AGCTTCTAGAGTTGGTCATATAGGAAGTTCTATAGATTTTGCTATTTTTCTCTTCATTT 360
OR906091|Cchi|En_M1 AGCTTCTAGAGTTGGTCATATAGGAAGTTCTATAGATTTTGCTATTTTTCTCTTCATTT 360
OR906092|Cchi|En_M2 AGCTTCTAGAGTTGGTCATATAGGAAGTTCTATAGATTTTGCTATTTTTCTCTTCATTT 360

OR906093|Csal|Ca_F  AGCTGGTGCTTCTTCTATTATAGGAGCCGTTAATTTTATTACTACCATTATCAATATACG 420
OR906095|Csal|V_F   AGCTGGTGCTTCTTCTATTATAGGAGCTGTTAATTTTATTACTACTATTATTAATATACG 420
KM225104.1|Csal|GF  AGCTGGTGCTTCTTCTATTATAGGAGCTGTTAATTTTATTACTACTATTATTAATATACG 420
ACG3675|Csal|Ho     AGCTGGGGCTTCTTCTATTATAGGAGCTGTAAATTTTATTACTACTATTATTAATATACG 420
OR906087|Cchi|Su_M  AGCTGGTGCTTCTTCTATTATAGGAGCTGTAAATTTTATTACTACTATTATTAATATGCG 420
OR906088|Cchi|Su_F  AGCTGGTGCTTCTTCTATTATAGGAGCTGTAAATTTTATTACTACTATTATTAATATGCG 420
OR906090|Cchi|En_F2 AGCTGGTGCTTCTTCTATTATAGGGGCTGTAAATTTTATTACTACTATTATTAATATGCG 420
OR906089|Cchi|En_F1 AGCTGGTGCTTCTTCTATTATAGGGGCTGTAAATTTTATTACTACTATTATTAATATGCG 420
OR906091|Cchi|En_M1 AGCTGGTGCTTCTTCTATTATAGGGGCTGTAAATTTTATTACTACTATTATCAATATGCG 420
OR906092|Cchi|En_M2 AGCTGGTGCTTCTTCTATTATAGGGGCTGTAAATTTTATTACTACTATTATTAATATGCG 420

OR906093|Csal|Ca_F  TTTATTAGGAATAAGAATAGAAAAAGTTCCATTATTTGTATGATCTGTATTTATTACTGC 480
OR906095|Csal|V_F   CTTATTAGGAATAAGAATAGAAAAAGTTCCATTGTTTGTATGATCTGTATTTATTACTGC 480
KM225104.1|Csal|GF  TTTATTAGGAATAAGAATAGAAAAAGTTCCATTATTTGTGTGATCTGTGTTTATTACTGC 480
ACG3675|Csal|Ho     TTTGGTAGGGATAAGAATAGAAAAAGTTCCATTATTTGTATGATCTGTATTTATTACTGC 480
OR906087|Cchi|Su_M  TTTGGTGGGAATAAGAATAGAAAAGGTTCCATTATTTGTATGATCTGTATTTATTACTGC 480
OR906088|Cchi|Su_F  TTTGGTGGGAATAAGAATAGAAAAGGTTCCATTATTTGTATGATCTGTATTTATTACTGC 480
OR906090|Cchi|En_F2 TTTGGTGGGAATAAGAATAGAAAAGGTTCCATTATTTGTATGATCTGTATTTATTACTGC 480
OR906089|Cchi|En_F1 TTTGGTGGGAATAAGAATAGAAAAGGTTCCATTATTTGTATGATCTGTATTTATTACTGC 480
OR906091|Cchi|En_M1 TTTGGTGGGAATAAGAATAGAAAAGGTTCCATTATTTGTATGATCTGTATTTATTACTGC 480
OR906092|Cchi|En_M2 TTTGGTGGGAATAAGAATAGAAAAGGTTCCATTATTTGTATGATCTGTATTTATTACTGC 480

OR906093|Csal|Ca_F  TATTTTATTATTATTATCTTTACCAGTTTTAGCTGGAGCTATTACTATATTATTAACTGA 540
OR906095|Csal|V_F   TATTTTATTATTATTATCTTTACCTGTTTTAGCAGGGGCTATTACTATATTATTAACTGA 540
KM225104.1|Csal|GF  TATTTTGTTATTATTATCTTTACCAGTTTTAGCTGGAGCTATTACTATATTATTAACTGA 540
ACG3675|Csal|Ho     TATTTTATTATTATTATCTTTACCAGTTTTAGCTGGAGCTATTACTATATTATTGACTGA 540
OR906087|Cchi|Su_M  TATTTTGTTATTATTATCTTTACCAGTTTTAGCTGGAGCTATCACTATATTGTTGACTGA 540
OR906088|Cchi|Su_F  TATTTTGTTATTATTATCTTTACCAGTTTTAGCTGGAGCTATTACTATATTGTTGACTGA 540
OR906090|Cchi|En_F2 TATTTTGTTATTATTATCTTTACCAGTTTTAGCTGGAGCTATTACTATATTGTTGACTGA 540
OR906089|Cchi|En_F1 TATTTTGTTATTATTATCTTTACCAGTTTTAGCTGGAGCTATTACTATATTGTTGACTGA 540
OR906091|Cchi|En_M1 TATTTTGTTATTATTATCTTTACCAGTTTTAGCTGGAGCTATTACTATATTGTTGACTGA 540
OR906092|Cchi|En_M2 TATTTTGTTAATATTATCTTTACCAGTTTTAGCTGGAGCTATTACTATATTGTTGACTGA 540
```

Cchi: *Cupiennius chiapanensis*; Csal: *Cupiennius salei*; En: Site - 1 La Encrucijada; Su: Site - 2 Suchiate; Ca: Site - 3 Cacahoatán; Ver: Site - 4 Los Tuxtlas; GF: French Guyana (KM225104.1); Ho: Honduras (BOLD:ACG3675); M: male; F: female.
